# Molecular Aspects of Cardiometabolic Diseases: From Etiopathogenesis to Potential Therapeutic Targets

**DOI:** 10.3390/ijms25115841

**Published:** 2024-05-27

**Authors:** Iveta Bernatova, Monika Bartekova

**Affiliations:** 1 Institute of Normal and Pathological Physiology, Centre of Experimental Medicine, Slovak Academy of Sciences, Sienkiewiczova 1, 81371 Bratislava, Slovakia; 2 Institute for Heart Research, Centre of Experimental Medicine, Slovak Academy of Sciences, 9 Dubravska cesta, 84104 Bratislava, Slovakia

Cardiometabolic diseases (CMDs) encompass a range of prevalent, often preventable, non-communicable illnesses, including myocardial infarction, stroke, cardiac insufficiency, arterial hypertension, obesity, type 2 diabetes mellitus, insulin resistance, chronic renal dysfunction, non-alcoholic fatty liver disease, and rare metabolic disorders [1]. As the leading health burden in the 21st century, the rise of CMDs is in line with the increasing prevalence of obesity and hypertension observed in both developing and industrialized countries [2,3,4,5,6].

Risk factors for CMDs are multifaceted, encompassing environmental influences, unhealthy dietary patterns, physical inactivity, stress, and epigenetic/genetic factors [7,8,9,10]. Similarly, the pathophysiology of CMDs is multifactorial, involving alterations in the sympathetic nervous system, renin-angiotensin-aldosterone system, endothelial dysfunction, inflammatory processes, and oxidative stress [11,12,13,14,15]. Additionally, defective genes, aberrant gene regulation, and alterations in intracellular and extracellular signaling pathways are involved in CMD development. Understanding these pathomechanisms, particularly the role of various nuclear factors and receptors, presents novel targets for CMD prevention and treatment.

This Special Issue features three original research articles and three review articles dedicated to enhancing our understanding of the mechanisms underlying CMDs and exploring preventive strategies for this group of non-communicable diseases.

The article by Tsigkou et al. (contribution 1) deals with the molecular mechanisms underlying the association of endothelial dysfunction with the development and progression of heart failure. The focus is on the activation of the sympathetic nervous system and its subsequent interaction with the renin-angiotensin-aldosterone system, oxidative stress, inflammation, nitric oxide and bradykinin release as well as calcium regulation and mitochondrial energetics. In addition, molecular mechanisms of endothelial dysfunction in patients with heart failure of various etiologies are summarized in this review along with the possibilities of prevention and treatment of endothelial dysfunction. These include non-pharmacological interventions including exercise training as well as the use of selected drugs such as statins, beta blockers, angiotensin-converting enzyme inhibitors and/or angiotensin receptor blockers, mineralocorticoid receptor antagonists, sodium-glucose cotransporter 2 inhibitors, an angiotensin-receptor/neprilysin inhibitor sacubitril-valsartan and endothelin receptor antagonists. The review shows that most of the therapeutic options with established benefits in patients with heart failure have a parallel beneficial effect in the restoration of endothelial function. A key role of the endothelium in the development of CMDs has also been identified in various preclinical studies and human studies [16,17,18].

In addition to the aforementioned factors, changes in the microbiota play an important role in the development of CMDs [19,20,21]. Indeed, a growing body of evidence suggests a bidirectional relationship between gut microbiota and nearly all known cardiovascular risk factors [22]. The article by Emilia Hijova (contribution 2) summarizes the current knowledge about the role of gut microbiota and metabolism in cardiovascular diseases (CVDs). It brings an overview of the potential beneficial effects of biotics (both probiotics and prebiotics) in preventing and treating CVDs. It also clearly points to the possibility of modulating the development of CMDs by specific beneficial metabolites derived from bacteria. It should also be mentioned that changes in gut bacteria and their metabolites are linked to age-related heart disease; manipulating the gut microbiome could be a novel therapeutic approach for preventing or treating this condition in older populations [23]. Thus, understanding the role of gut microbiota in the development of CVDs might open new roads of cardioprotection through dietary supplements called biotics.

A contribution by Lee et al. (contribution 3) brought very interesting data documenting that a ketogenic diet (KD) may also contribute to cardioprotection, particularly that KD may prevent dysregulation in Na^+^ and Ca^2+^ homeostasis induced by diabetes mellitus. The cardioprotective potential of KD has been recently proposed by several researchers, but the authors significantly contributed to the knowledge in the field, particularly in diabetic subjects [24,25,26,27]. They have shown in an animal model of streptozotocin-induced diabetes that six-week KD led to improved ECGs of the hearts of diabetic rats. Moreover, KD improved intracellular Ca^2+^ transients, sarcoplasmic reticular Ca^2+^ content, sodium (Na^+^)-Ca^2+^ exchanger currents, L-type Ca^2+^ content, and sarcoplasmic reticulum ATPase content. KD also normalized the ratios of phosphorylated-to-total proteins across Ca^2+^-handling proteins, including ryanodine receptor 2, phospholamban, and calmodulin-dependent protein kinase II. KD also reduced the incidence of Ca^2+^ leaks and levels of cytosolic reactive oxygen species in the hearts of diabetic rats. Thus, KD may represent a powerful dietetic approach for the prevention and treatment of diabetic cardiomyopathy, which significantly contributes to the overall knowledge about the positive effects of KD on cells [28,29].

The study of Law et al. (contribution 4) conducted untargeted lipidomic investigations to assess the alterations of lipid profiles in an apolipoprotein E knockout (apoE^−/−^) mouse model, with or without feeding a high-fat diet aimed to potentially uncover alternative therapeutic approaches other than cholesterol- and triglyceride-lowering medications to treat atherosclerotic cardiovascular disease [30,31,32]. They have shown that in addition to hypercholesterolemia and hyperlipidemia, levels of lipid moieties including lysophosphatidylcholine, sphingomyelin, and ceramide were two to five times higher in apoE^−/−^ mice compared to wild-type mice, and these levels were even higher due to a high-fat diet. Thus, lipid moieties may contribute to the early onset of atherosclerosis in apoE^−/−^ mice, and high-fat diet-fed apoE^−/−^ mice seem to be a suitable model for developing lysophosphatidylcholine- and ceramide-lowering therapies for the treatment of cardiometabolic diseases [33,34,35,36].

The original research article by Liskova et al. (contribution 5) investigated the effects of the natural flavonol taxifolin, also known as dihydroquercetin. Cardioprotective effects of taxifolin were found also against diazinon-induced myocardial injury in rats and against cardiac hypertrophy and fibrosis during biomechanical stress of pressure overload [37,38]. Liskova et al. demonstrated that a 10-day taxifolin treatment lowered blood pressure in hypertensive rats. Taxifolin improved endothelium-dependent relaxation and reduced endothelium-dependent contraction in femoral arteries. Taxifolin increased total nitric oxide synthase activity and anti-inflammatory interleukin-10 expression, while decreasing cyclooxygenase-2 expression, suggesting complex effects on blood pressure reduction. In addition, using in vitro experiments, the authors stated that the therapeutic benefits of taxifolin treatment for hypertensive individuals may result from taxifolin’s anti-inflammatory action in vasculature rather than from its antioxidant properties, which were not confirmed in their in vivo experiments.

Another contribution in this Special Issue was focused on the role of AMP-dependent protein kinase (AMPK), metabolic stress sensors, which contribute to stress resilience and extended lifespan in lower model organisms [39,40]. Kvandova et al. (contribution 6) provide an overview of the current knowledge on AMPK, its structure, functions, activation, interaction with sex hormones, and its effects on endothelial function cardiometabolic health. AMPK, with its antioxidant, anti-inflammatory, and metabolic effects, interacts with sex hormones impacting CMD development. Thus, the authors conclude that AMPK provides the potential for targeted therapies based on sex hormones, making it a compelling area for future research.

All articles featured within this Special Issue presented novel and valuable insights into the mechanisms underlying the development and progression of CMDs, along with their prevention and/or treatment. As Guest Editors of this Special Issue, we would like to express our sincere gratitude to all the contributing authors whose work, including both original experimental studies and comprehensive literature reviews, has enriched this Special Issue. We believe that this Special Issue represents new information for researchers engaged in molecular mechanisms associated with CMDs, as well as the prevention and treatment of these non-communicable diseases.
